# A Deep-Network Piecewise Linear Approximation Formula

**DOI:** 10.1109/access.2021.3109173

**Published:** 2021-08-31

**Authors:** GENGSHENG L. ZENG

**Affiliations:** Department of Computer Science, Utah Valley University, Orem, UT 84058, USA; Department of Radiology and Imaging Sciences, The University of Utah, Salt Lake City, UT 84108, USA

**Keywords:** Approximation algorithms, artificial neural networks, function approximation, interpolation, multi-layer neural network

## Abstract

The mathematical foundation of deep learning is the theorem that any continuous function can be approximated within any specified accuracy by using a neural network with certain non-linear activation functions. However, this theorem does not tell us what the network architecture should be and what the values of the weights are. One must train the network to estimate the weights. There is no guarantee that the optimal weights can be reached after training. This paper develops an explicit architecture of a universal deep network by using the Gray code order and develops an explicit formula for the weights of this deep network. This architecture is target function independent. Once the target function is known, the weights are calculated by the proposed formula, and no training is required. There is no concern whether the training may or may not reach the optimal weights. This deep network gives the same result as the shallow piecewise linear interpolation function for an arbitrary target function.

## I. INTRODUCTION

As suggested in [1], the idea of using a deep neural network can be traced all the way back to 1962 by Rosenblatt’s study on neuro-dynamics. Rosenblatt simulated Mark I Perceptron on an IBM computer. Unfortunately, the mainstream research on neural network slowly started after his death in 1971. An early deep learning network algorithm was developed by Ivakhnenko and Lapa in 1967 [2]. They described a deep learning net with 8 layers. Their method was complicated. Given a training set of input vectors with corresponding target output vectors, layers of additive and multiplicative neuron-like nodes were incrementally grown and trained by regression analysis, then pruned with the help of a separate validation set, where regularization was used to weed out superfluous nodes. The numbers of layers and nodes per layer were learned in problem-dependent fashion.

The breakthrough in deep learning was the development of the backpropagation algorithm by Rumelhart *et al*. [3]. The backpropagation algorithm is essentially the chain rule in calculating the derivatives in calculus, which makes deep neuro network training efficient. In 1989, LeCun *et al*. [4] applied the standard backpropagation algorithm to a deep neural network. Their application in [4] was to recognize handwritten ZIP codes on mail. In 2009, Hinton developed deep belief networks [5]. These modern-day deep networks had outstanding performances.

In addition to the breakthrough in algorithm development, advances in hardware have made deep learning more efficient. In 2009, Nvidia was involved in what was called the “big bang” of deep learning; graphics processing units (GPUs) increased the speed of deep learning more than 10 times [6]. The availability of huge amount of data makes machine learning practical [7]. It seems that the theory lags behind practices in the field of deep learning. It is still not very clear why and how the deep networks work so well.

The mathematical theory of artificial neural networks is the universal approximation theorem [8]. The essence of this theorem implies that on a finite interval, any continuous function can be accurately approximated by a neural network with nonlinear activation functions. In 2020, Sci *et al*. [9] showed that the minimal number of 2 hidden layers are necessary and sufficient for any *d*-dimensional (*d*-D, *d* > 1) continuous piecewise linear function to be represented by a ReLU deep neural network (DNN). The ReLU function, *σ*(*x*) = max(0, *x*), is a popular nonlinear activation function. When *d* = 1, only 1 hidden layer is required. When a shallow network can do the job, why do people use deep networks?

In 2017, Yarotsky [10] compared the shallow networks with deep networks and concluded that the deep networks may have less complexity than the shallow networks. The complexity of networks is measured by the number of the weights and computation units [11]. In other words, the advantage of using a deep network over using a shallow network is that we may train fewer weights and reach the same approximation accuracy.

All these theoretical results do not indicate what deep network architecture we should choose to approximate a target function. There is no guarantee that the weights will converge to the optimal solution after training.

The main contribution of this paper is to construct a universal deep network for piecewise linear interpolation of any continuous target function with a specified accuracy. By universal, we mean that the architecture of the deep network is the same for all target functions. The specified accuracy dictates the depth of the network. The weights of the network are calculated explicitly by a formula, instead of being trained by input/output data pairs.

One way to obtain a shallow network approximation of a target function is to use the first order spline interpolation, or piecewise linear interpolation [12]. The formula for each line segment in the interpolation is determined by the values of the 2 endpoints of the segment. The same piecewise linear interpolation function can also be realized by a deep network (instead of a shallow network). This paper will develop an explicit architecture of this universal deep network by using the Gray code order [13] and will develop a formula for the weights of this deep network.

This paper gives an explicit universal deep network for any given target function. The approximation accuracy is guaranteed to achieve the specified accuracy. The weights of the network are calculated by an explicit formula. No training is required. The drawback of our proposed deep network is that it may not be the most efficient network to approximate a given target function.

## II. METHODS

In this paper, we develop a formula for the weights of a universal deep network. This network performs piecewise-linear approximation of a one-dimensional (1D) continuous target function *f* (*x*) on [*a*, *b*]. We also extend this deep network to the situations where the target function is *d*-dimensional (*d*-D). Without loss of generality, let [*a*, *b*] = [0, 1]. We consider a deep network with *N* hidden layers. We uniformly sample the target function *f* (*x*) at 2*^N^* + 1 points, called knots. The knots are 0/2*^N^*, 1/2*^N^*, 2/2*^N^*, 3/2*^N^*,…, 2*^N^*/2*^N^*.

### A. LINEAR INTERPOLATION FORMULA FOR 1D FUNCTIONS

Let the 1D continuous target function on [0, 1] be *f* (*x*), for the uniformly distributed knots 0/2*^N^*, 1/2*^N^*, 2/2*^N^*, 3/2*^n^* ,…, 2*^N^* /2*^N^* , if *f* (0) = *f* (1) = 0, the linear interpolation formula is given as [12]

(1) $$f\;(x)\approx \hat{f}\;(x)=\sum_{m=1}^{2^{N}-1}f\left(\frac{m}{2^{N}}\right)y_{m,N}\;(x),$$

**FIGURE 1.**
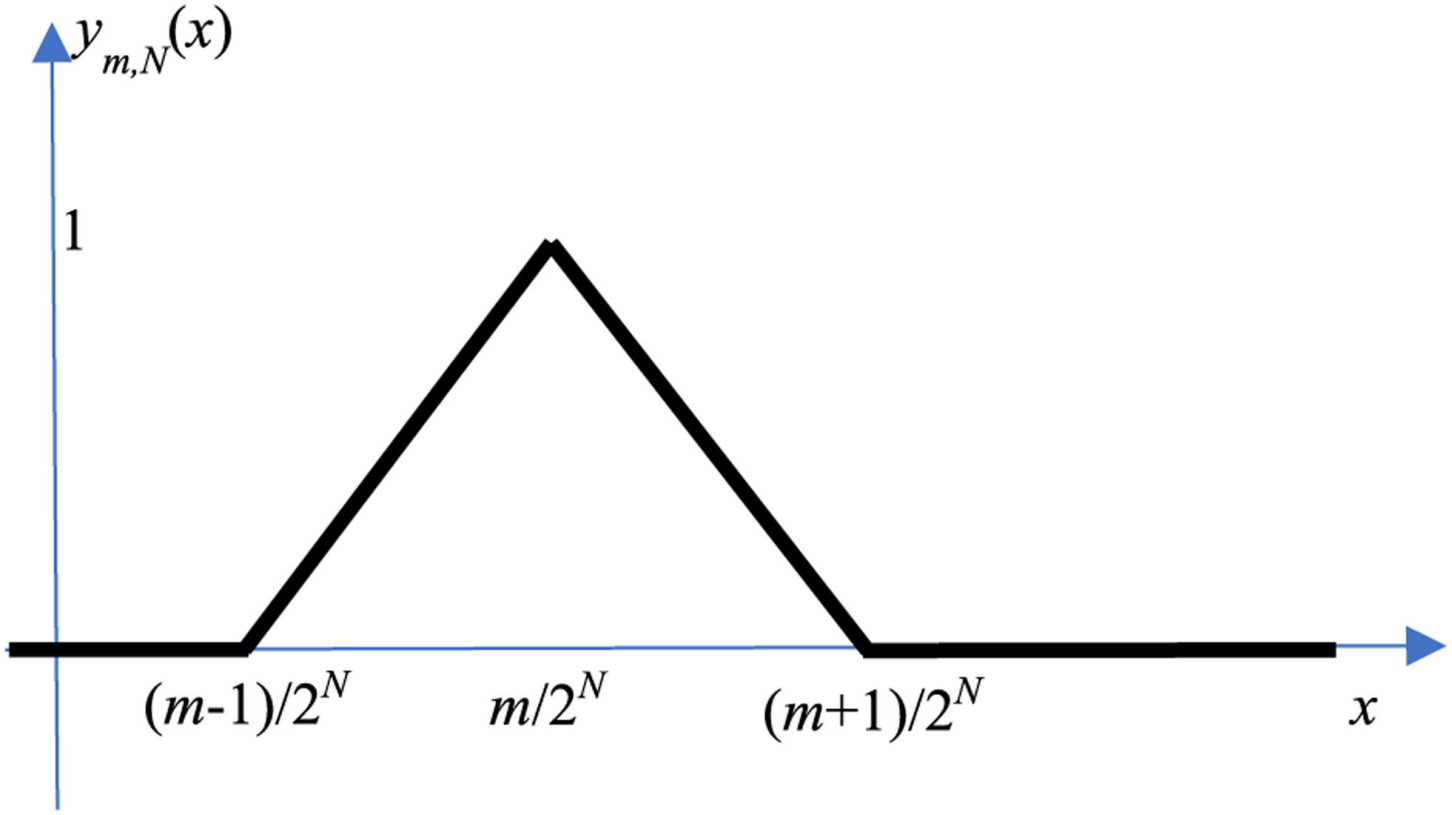
The hat function *y_m,N_*(*x*).

Where

(2) $$y_{m,N}\;(x)=2^{N}[\sigma \left(x-\frac{m-1}{2^{N}}\right)-2\sigma \left(x-\frac{m}{2^{N}}\right)\;\;+\sigma \left(x-\frac{m+1}{2^{N}}\right)]$$

is a triangular function (or hat function) expressed by the ReLU functions. The triangular function *y_m_*,*_N_*(*x*) is illustrated in Fig. 1. In engineering, the linear interpolation formula (1) is also referred to as the first order hold [14].

If the equations *f* (0) = *f* (1) = 0 do not hold, (1) can be modified to

(3) $$\hat{f}\;(x)=\sum_{m=1}^{2^{N}-1}f\left(\frac{m}{2^{N}}\right)y_{m,N}\;(x)+[f\;(1)-f\;(0)]x+f\;(0).$$

Formula (3) can be directly used to construct a shallow network to approximate the 1D target function *f*(*x*). The hyper parameter *N* is determined by the target function *f*(*x*) and the approximation accuracy requirement.

### B. CONSTRUCTION OF A 1D DEEP NETWORK WITH 3 HIDDEN LAYERS

Let us introduce two neuron functions *g*_0_(*x*) and *g*_1_(*x*), which are two non-linear functions defined as

(4) g0(x)={2xif0≤x<0.50otherwise}

and

**FIGURE 2.**
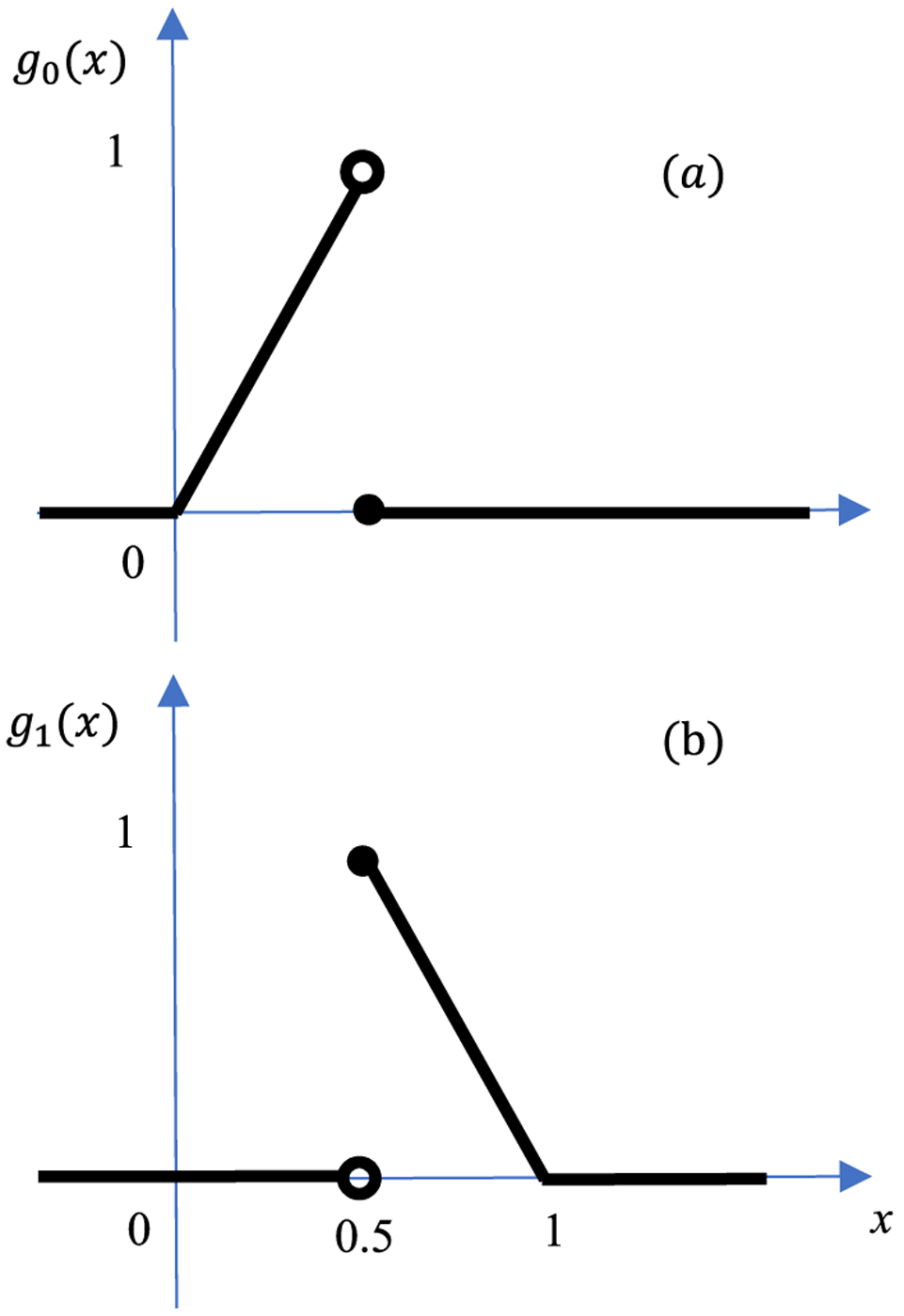
The left neuron *g*_0_ (*x*) shown in (a) and the right neuron *g*_1_ (*x*) shown in (b) are two non-linear functions.

(5) g1(x)={−2(x−1)if0.5≤x<10otherwise}

respectively. These two functions are depicted in Fig. 2 (a) and (b), respectively. The sum *g*_0_(*x*) + *g*_1_(*x*) forms the well-known ‘hat’ function or the ‘triangle’ function, *y*_1,1_, commonly used as the kernel function for linear interpolation.

**FIGURE 3.**
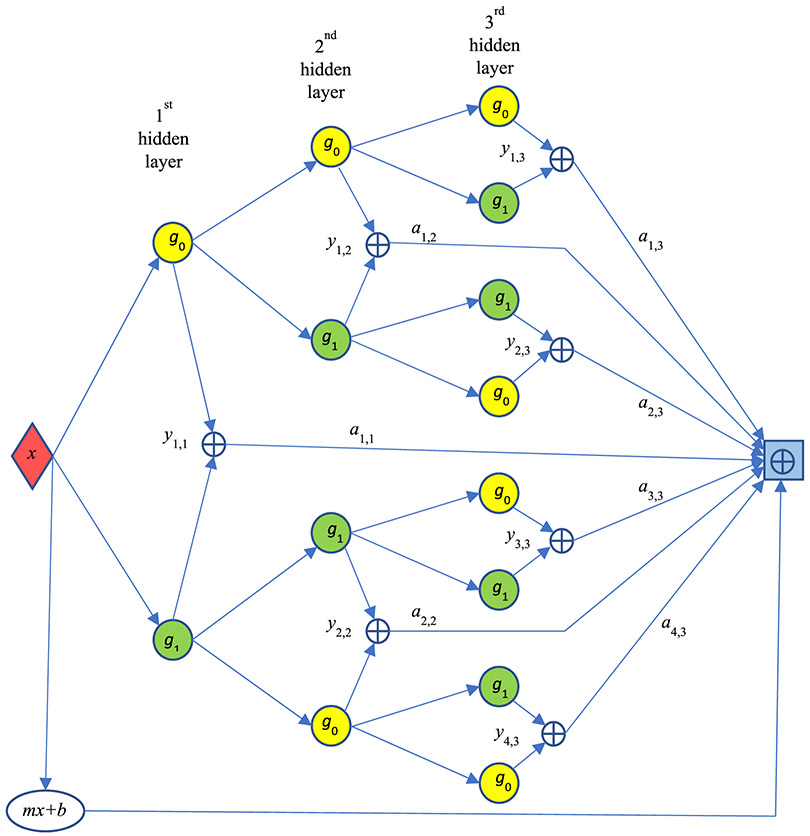
The proposed deep network uses a piecewise linear function to approximate a general target function *f* (*x*). Three hidden layers are used in this example.

Let us construct a deep-network layer by layer. See Fig. 3 for the deep network architecture, which looks like a binary tree. In this particular example, there are 5 layers: 1 input layer, 3 hidden layers, and 1 output layer. The input layer consists of only one unit (as a red diamond), representing the function variable *x*.

The 1^st^ hidden layer has two units, one being the ‘left’ neuron *g*_0_ (*x*) and the other being the ‘right’ neuron *g*_1_ (*x*). The input of these two units is the variable *x*. The output of the left neuron is *g*_0_ (*x*), and the output of the ‘right’ neuron *g*_1_ (*x*). In Fig. 3, the left neuron is represented by a yellow circle, and the right neuron is a green circle.

There are 4 units in the 2^nd^ hidden layer. The output from each unit at the previous layer is connected to two units: a left (L) neuron and a right (R) neuron. The input of the bottom unit in the 2^nd^ hidden layer is *g*_1_ (*x*), and the output of this unit is *g*_0_ (*g*_1_ (*x*)). Please pay attention to the order of left and right neurons. They are *not* in the alternating L-R order. From top to bottom in Fig. 3, the order is: L-R, R-L, L-R, R-L, and so on.

There are 8 = 2^3^ units in the 3^rd^ hidden layer. In fact, there are 2*^N^* units in the *N*^th^ hidden layer. The input of the bottom unit in the 3^rd^ hidden layer is *g*_0_ (*g*_1_ (*x*)), and the output of this unit is *g*_0_(*g*_0_(*g*_1_(*x*))). The arrangement of the hidden neurons follows the Gray code order, which is listed in Table 1. For the example of 3 hidden layers as in Fig. 3, the subscripts of the neuron functions follow exactly the order of the 3-bit Gray code shown in the 3^rd^ column of Table 1. For the example of 2 hidden layers, the subscripts of the neuron functions follow the order of the 2-bit Gray code. In general, if we want to construct a deep network for function approximation, we can readily obtain the architecture according to the order of the N-bit Gray code.

If we implement the proposed deep network and ignore the required Gray code order, the resultant hat functions at each layer’s adder outputs may be in undesired locations. For example, the second hat function may appear at the location of the third hat function.

As shown in Fig. 3, the output layer is a summation unit without any nonlinearity. The final output is the piecewise linearly approximated function to the given target function *f* (*x*) and is evaluated as the weighted sum of the outputs at every hidden layers. In a common deep network design, the weighting factors *a_j,i_* are trained by many input/output pairs. In this paper, the weighting factors *a_j,i_* are calculated by a formula. The general form of this formula is given in Eq. (24) later in this section. No training is needed.

**FIGURE 4.**
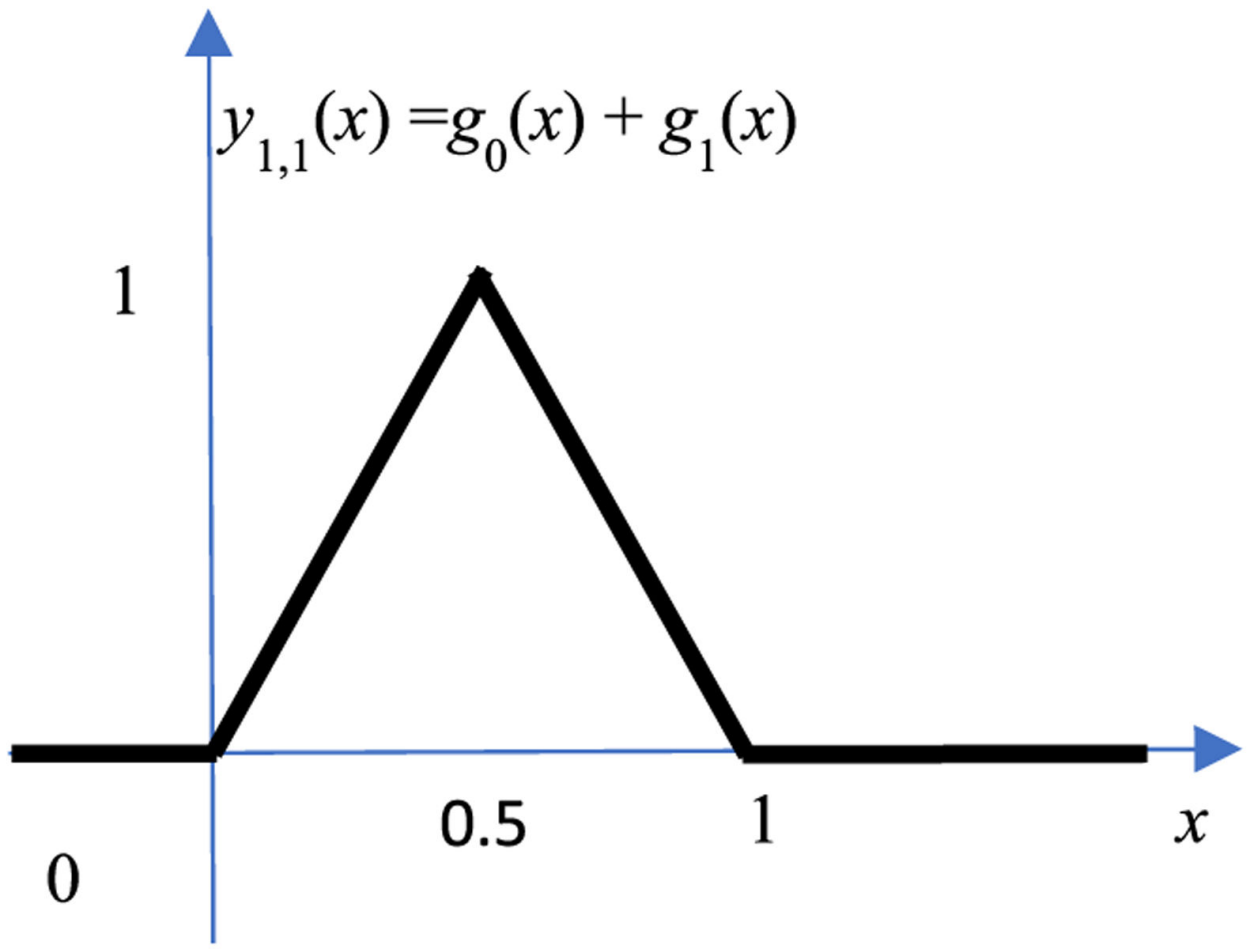
The hat function *y*_1,1_ (*x*) = *g*_0_ (*x*) + *g*_1_ (*x*).

**FIGURE 5.**
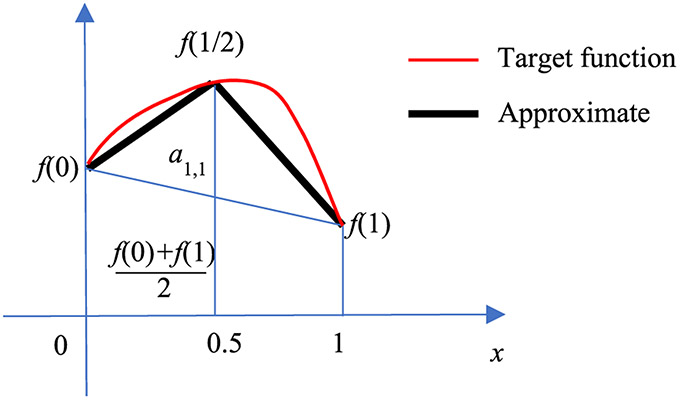
Using a hat function *y*_1,1_ (*x*) (black) to approximate a target function *f* (*x*) (red) on [0, 1].

Let us first consider the adder output *y*_1,1_ (*x*) from the 1^st^ hidden layer adder. This adder sums the functions *y*_1,1_ (*x*) = *g*_0_ (*x*) + *g*_1_ (*x*). Therefore, the output is a hat function as shown in Fig. 4. Fig. 5 illustrates how a hat function is used to approximate a general function *f* (*x*) on [0, 1]. In Fig. 5, the red curve represents the target function *f* (*x*). The black curve is the piecewise approximation $\hat{f}\;(x)$, which is the summation of a linear function *mx*+*b* and a hat function *a*_1,1_ *y*_1,1_ (*x*):

(6) $$\hat{f}\;(x)=mx+b+a_{1,1}y_{1,1}\;(x),$$

where

(7) m=f(1)−f(0),

(8) b=f(0),

(9) $$a_{1,1}=f\left(\frac{1}{2}\right)-\frac{f\left(\frac{0}{2}\right)+f\left(\frac{2}{2}\right)}{2}.$$

The purpose of the linear function *mx* + *b* (which is shown at the lower-left corner of Fig. 3) is to make the hidden layers to approximate a function that satisfies *f* (0) = *f* (1) = 0, because the two endpoints of a hat function have a value of 0 and the hidden layers assume that the endpoints must be 0.

In Fig. 5, there is only one knot between 0 and 1. The approximation is rather poor. To improve the approximation, we need add a 2^nd^ hidden layer and use 2 more knots at 1/4 and 3/4, respectively. It is straightforward to verify that the adder outputs of the 2^nd^ hidden layers are

(10) $$y_{1,2}\;(x)=g_{0}\;(g_{0}\;(x))+g_{1}\;(g_{0}\;(x)),$$

(11) $$y_{2,2}\;(x)=g_{1}\;(g_{1}\;(x))+g_{0}\;(g_{1}\;(x)).$$

**FIGURE 6.**
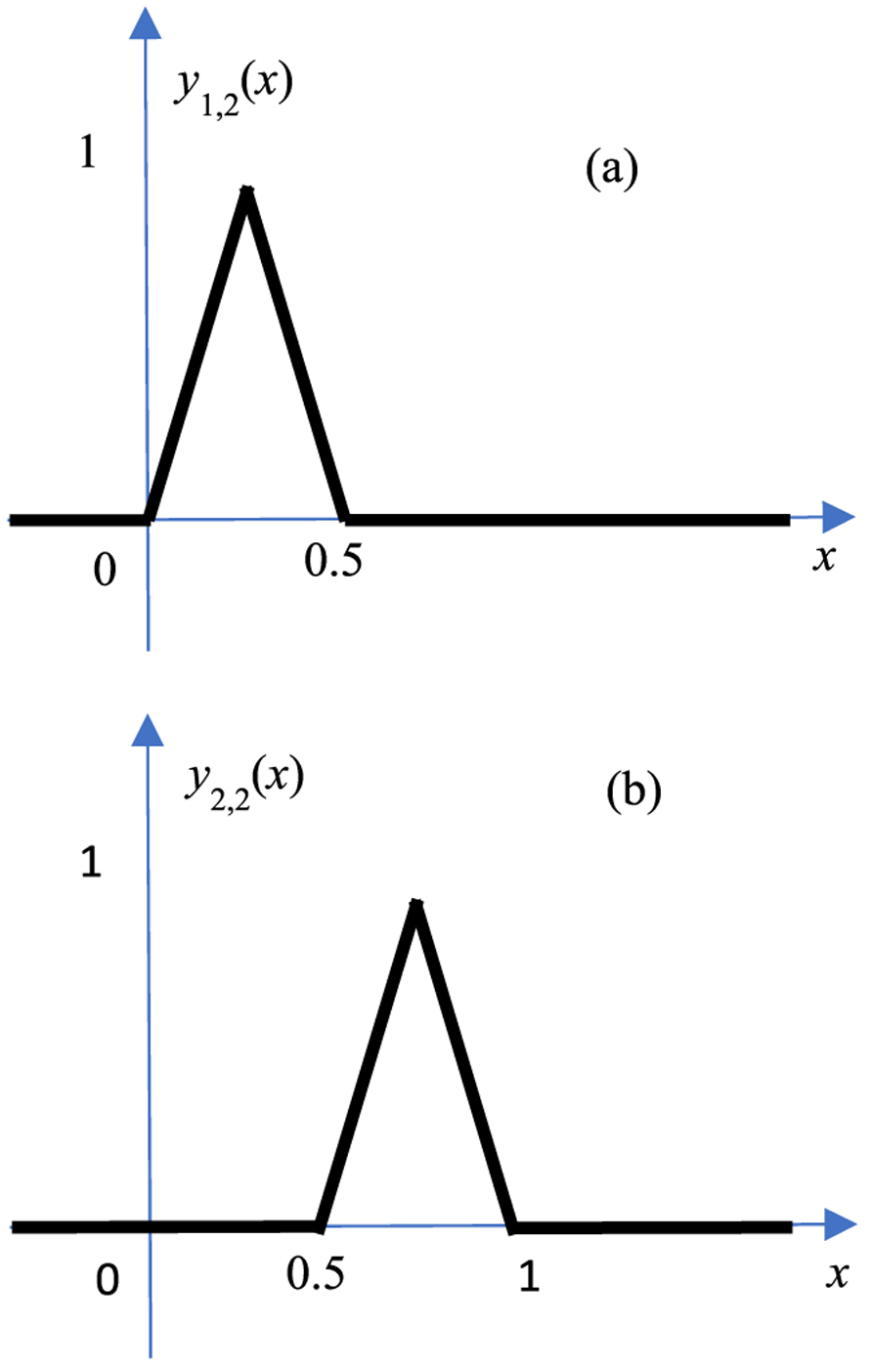
The hat functions *y*_1,2_(*x*) as shown in (a) and *y*_2,2_(*x*) as shown in (b).

The functions *y*_1,2_ (*x*) and *y*_2,2_ (*x*) are 2 different hat functions, with half the width as the width of *y*_1,1_ (*x*). These 2 hat functions are at different locations as shown in Fig. 6. With the additional 2 knots at 1/4 and 3/4, the approximation (6) is improved to

(12) $$\hat{f}\;(x)=mx+b+a_{1,1}y_{1,1}\;(x)+a_{1,2}y_{1,2}\;(x)+a_{2,2}y_{2,2}\;(x)$$

Where

(13) $$a_{1,2}=f\left(\frac{1}{4}\right)-\frac{f\left(\frac{0}{4}\right)+f\left(\frac{2}{4}\right)}{2}$$

(14) $$a_{2,2}=f\left(\frac{3}{4}\right)-\frac{f\left(\frac{2}{4}\right)+f\left(\frac{4}{4}\right)}{2}.$$

**FIGURE 7.**
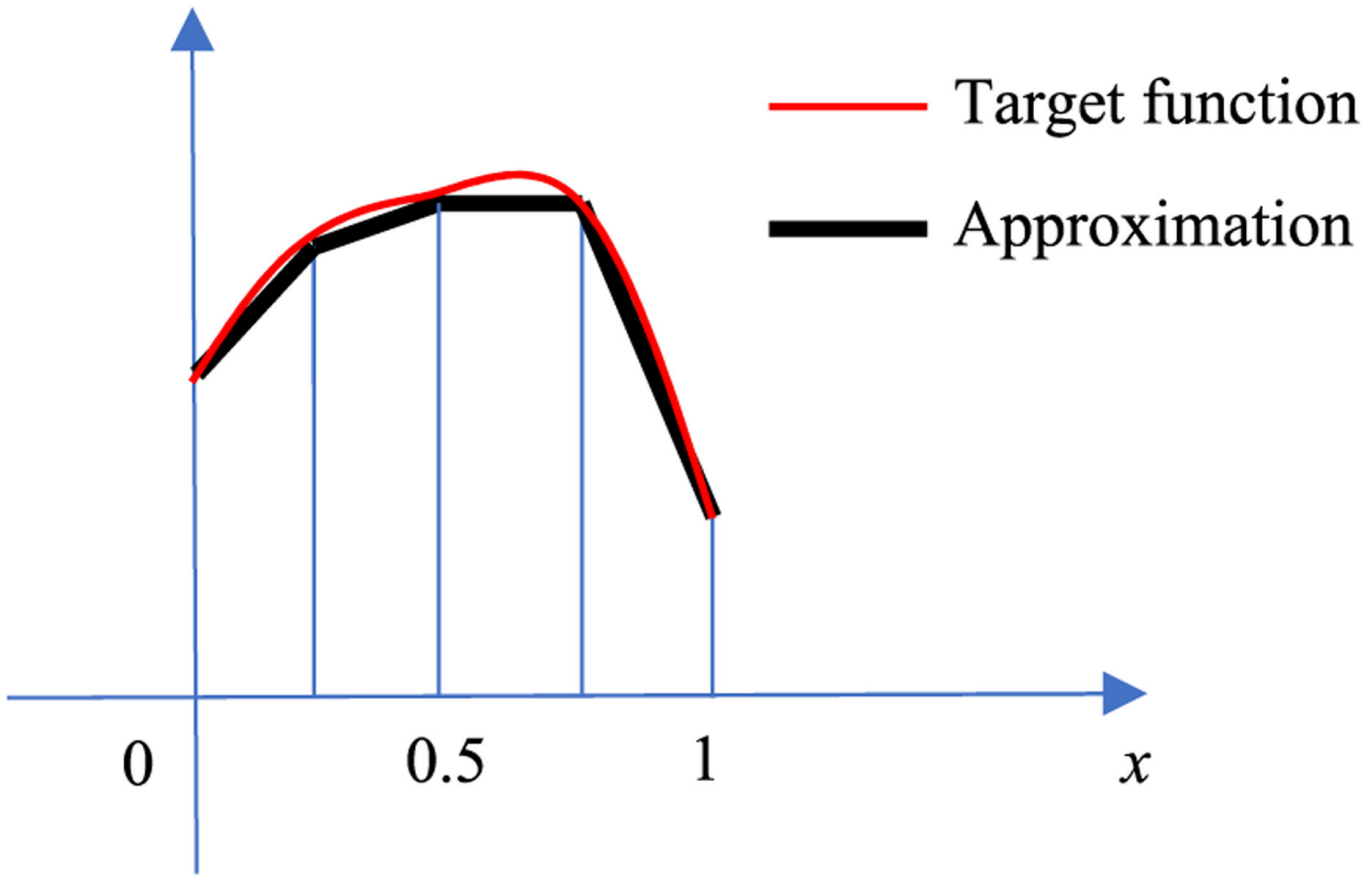
The piecewise linear function (black) using hat functions *y*_1,1_(*x*), *y*_1,2_(*x*) and *y*_2,2_(*x*) to approximate a target function *f* (*x*) (red) on [0, 1].

The improvement is illustrated in Fig. 7, where the original target function *f* (*x*) (in red) and the piecewise approximation $\hat{f}\;(x)$ (in black) are equal at knots 0, 1/4, 1/2, 3/4, and 1.

To improve the approximation further, we can add a 3^rd^ hidden layer and use 4 more knots at 1/8, 3/8, 5/8 and 7/8. It can be verified that the adder outputs of the 3^rd^ hidden layers are

(15) $$y_{1,3}\;(x)=g_{0}\;(g_{0}\;(g_{0}\;(x)))+g_{1}\;(g_{0}\;(g_{0}\;(x))),$$

(16) $$y_{2,3}\;(x)=g_{1}\;(g_{1}\;(g_{0}\;(x)))+g_{0}\;(g_{1}\;(g_{0}\;(x))),$$

(17) $$y_{3,3}\;(x)=g_{0}\;(g_{1}\;(g_{1}\;(x)))+g_{1}\;(g_{1}\;(g_{1}\;(x))),$$

(18) $$y_{4,3}\;(x)=g_{1}\;(g_{0}\;(g_{1}\;(x)))+g_{0}\;(g_{0}\;(g_{1}\;(x))).$$

**FIGURE 8.**
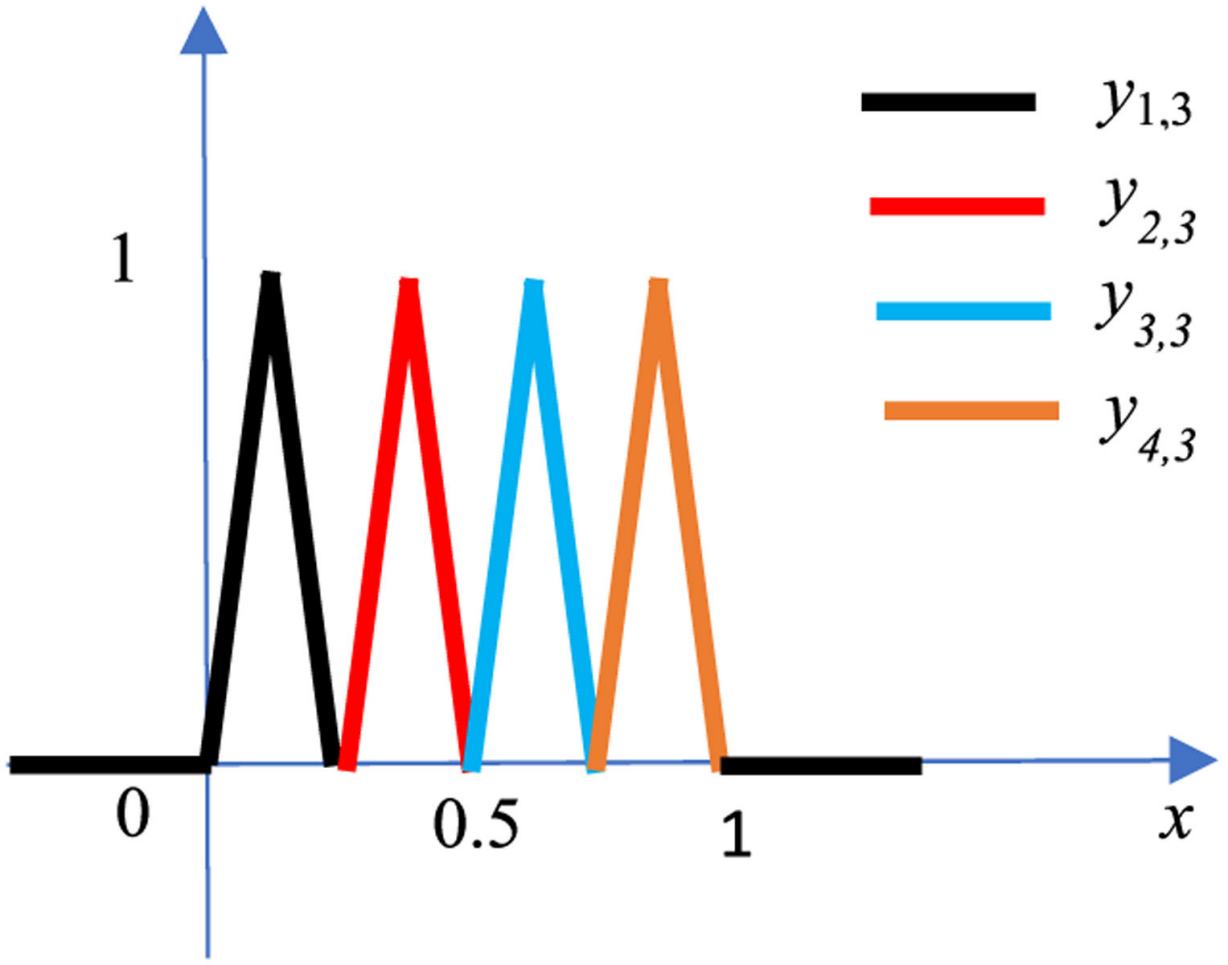
The 4 hat functions in the 3^rd^ layer: *y*_1,3_(*x*) in black, *y*_2,3_(*x*) in red, *y*_3,3_(*x*) in blue and *y*_4,3_(*x*) in orange.

The functions in (15)-(18) are 4 different hat functions, each with a width of 1/4. These 4 hat functions are at different location as shown in Fig. 8. With the additional 4 knots, the approximation (12) is improved to

(19) $$\hat{f}\;(x)=mx+b+a_{1,1}y_{1,1}\;(x)+a_{1,2}y_{1,2}\;(x)+a_{2,2}y_{2,2}\;(x)\;\;+a_{1,3}y_{1,3}\;(x)+a_{2,3}y_{2,3}\;(x)+a_{3,3}y_{3,3}\;(x)\;\;+a_{4,3}y_{4,3}\;(x)$$

Where

(20) $$a_{1,3}=f\left(\frac{1}{8}\right)-\frac{f\left(\frac{0}{8}\right)+f\left(\frac{2}{8}\right)}{2},$$

(21) $$a_{2,3}=f\left(\frac{3}{8}\right)-\frac{f\left(\frac{2}{8}\right)+f\left(\frac{4}{8}\right)}{2},$$

(22) $$a_{3,3}=f\left(\frac{5}{8}\right)-\frac{f\left(\frac{4}{8}\right)+f\left(\frac{6}{8}\right)}{2},$$

(23) $$a_{4,3}=f\left(\frac{7}{8}\right)-\frac{f\left(\frac{6}{8}\right)+f\left(\frac{8}{8}\right)}{2}.$$

### C. CONSTRUCTION OF A 1D DEEP NETWORK WITH N HIDDEN LAYERS

In general, we construct a universal deep network with *N* hidden layers to approximate the target function *f* (*x*) with a piecewise linear approximation $\hat{f}\;(x)$ such that they are equal at 2*^N^* + 1 equally distributed knots: 0/2*^N^* , 1/2*^N^*, 2/2*^N^* , 3/2*^N^* ,…, (2*^N^* – 1) /2*^N^*, 2*^N^*/2*^N^*. The input layer and the hidden layers form a binary tree architecture, with the input layer being the root of the binary tree.

In a binary tree, each node has two children, which are referred to as the left child and the right child. In our case, the left child is not always the left neuron. Likewise, the right child is not always the right neuron. The location of a left neuron *g*_0_ (*x*) and a right neuron *g*_1_ (*x*) must be assigned according to the Gray code order as shown in Table 1.

At each layer, the order is the order is: L-R, R-L, L-R, R-L, and so on. There is an adder for each pair of left and right neurons, and the output of an adder is labeled as *y_j,i_* (*x*), where *i* is the hidden layer index and *j* is the hat function index. Here, *y_j,i_*, (*x*) is a hat function of width 1/2^*i*−1^, height 1, and support [(*j* − 1) /2^*i*−1^, (*j* + 1)/2^*i*−1^]. For a fixed *i*, there are 2^*i*−1^ hat functions *y_j,i_* (*x*), *j* = 1, 2, …, 2^*i*−1^, and the supports of them partition the interval [0, 1], similar to the illustration in Fig. 8.

**FIGURE 9.**
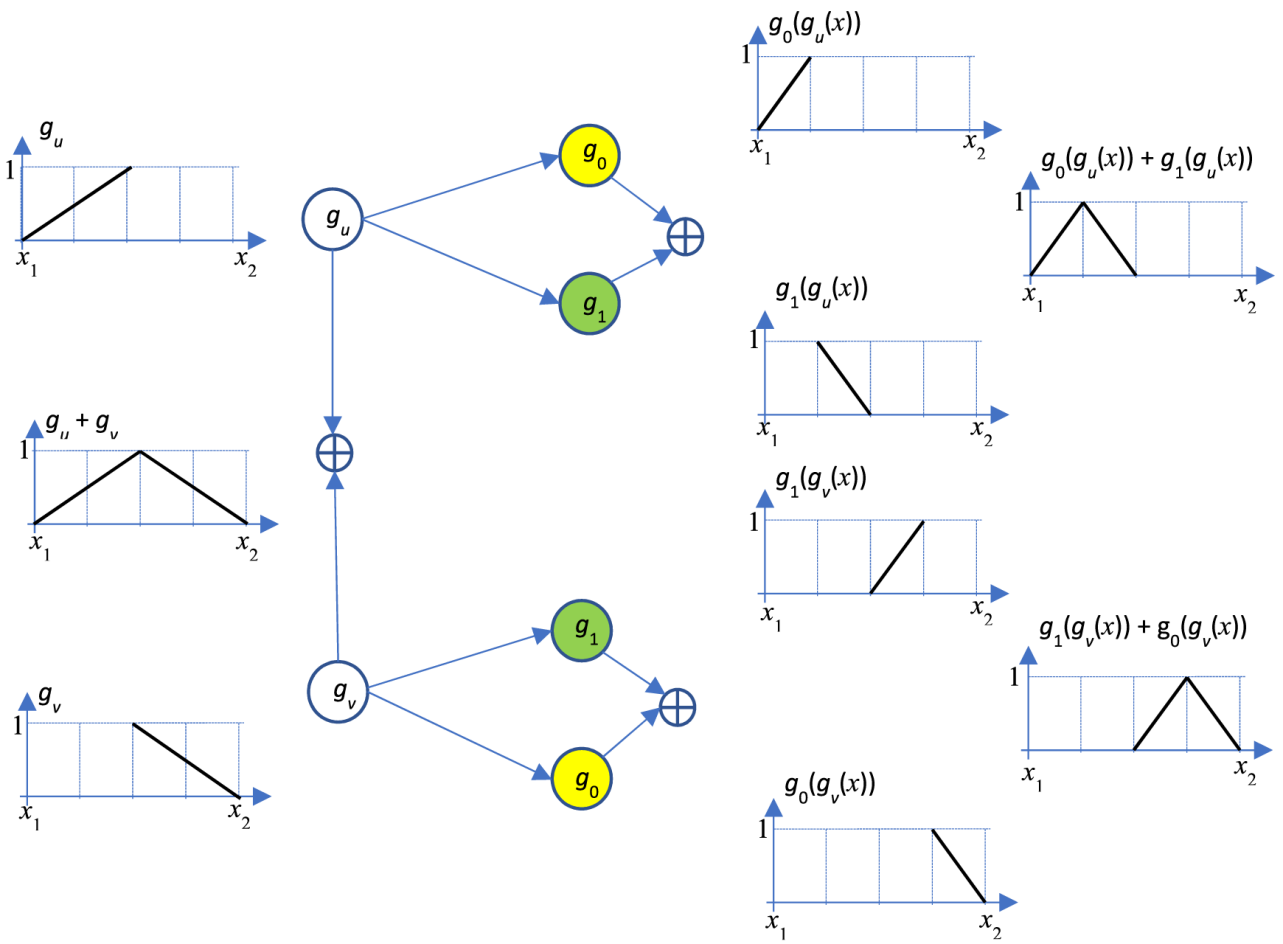
A general layer-to-layer relationship. The end effects are that the width of the hat function is halved, and the number of hat functions is doubled.

Now, let us consider the composition effects of the neuron functions: *g*_0_ (*x*) and *g*_1_ (*x*). The left neuron *g*_0_ (*x*) linearly maps [0, 1/2) to [0, 1); the right neuron *g*_1_ (*x*) linearly maps [1/2, 1] to [0, 1]. The relationship between two adjacent hidden layers is explained in Fig. 9, where *g_u_* (*x*) and *g_v_* (*x*) are the composed functions from the input layer to the current layer. The sum *g_u_* (*x*) + *g_v_* (*x*) forms a hat function; the width of this hat function is *x*_2_ − *x*_1_. To form the next layer, a left/right neuron pair is connected the outputs of *g_u_* (*x*) and *g_v_* (*x*), respectively. Using the definition of these two neuron functions: *g*_0_ (*x*) and *g*_1_ (*x*), in (4) and (5), respectively, the output functions of the next layer are obtained and shown in Fig. 9. The end effects are that the width of the hat function is halved, and the number of hat functions is doubled.

The main result of this paper is the general formula for the weights at the *i*th hidden layer, which is given as

(24) $$a_{j,i}=f\left(\frac{2j-1}{2^{i}}\right)-\frac{f\left(\frac{2j-2}{2^{i}}\right)+f\left(\frac{2j}{2^{i}}\right)}{2}$$

for *j* = 1, 2, 3,…, 2^*i*−1^, and *i* = 1, 2, 3,…, *N*. The final expression of the approximation function is given by

(25) $$\hat{f}\;(x)=mx+b+\sum_{i=1}^{N}\sum_{j=1}^{2^{i-1}}a_{j,i}y_{j,i}\;(x)\;\;=[f\;(1)-f\;(0)]x+f\;(0)+\sum_{i=1}^{N}\sum_{j=1}^{2^{i-1}}a_{j,i}y_{j,i}\;(x)$$

where *y*_j,i_ (*x*) is the hat function defined by

(26) yj,i(x)=2i[σ(x−2j−22i)−2σ(x−2j−12i)]+[σ(x−2j2i)]

with the ReLU function *σ* (*x*) = max (0, *x*), for *j* = 1, 2, 3, …, 2^*i*−1^, and i = 1, 2, 3,…, *N*. It can be readily verified that the deep network approximation (25) is the same as the shallow network approximation (3) if they use the same number of knots. In fact, these two approximations have the same values at the sampling knots as the target function and they take the linear interpolated values between the knots.

### D. EXTENSION TO A 2D DEEP NETWORK WITH N HIDDEN LAYERS

If the target function is two-dimensional (2D), *f* (*x*_1_, *x*_2_), its deep network approximation), $\hat{f}(x_{1},x_{2})$, can be obtained by using the results from the 1D case presented in Part C.

Let *f* (*x*_1_, *x*_2_) be sampled uniformly at the knots (*m*/2*^N^*, n/2*^N^*) with *n*, *m* = 0, 1, 2,…, 2*^N^*. When the index *n* is fixed, *f* (*x*_1_, *n*/2*^N^*) is a 1D function with the variable *x*_1_.

Using (25) for *n* = 0, 1 , 2, … , 2*^N^* , we have

(27) $$\hat{f}\left(x_{1},\frac{n}{2^{N}}\right)=\left[f\left(1,\frac{n}{2^{N}}\right)-f\left(0,\frac{n}{2^{N}}\right)\right]x_{1}+f\left(0,\frac{n}{2^{N}}\right)\;\;+\sum_{i=1}^{N}\sum_{j=1}^{2^{i-1}}a_{j,i}(\frac{n}{2^{N}})y_{j,i}\;(x_{1})$$

where

(28) $$a_{j,i}\left(\frac{n}{2^{N}}\right)=f\left(\frac{2j-1}{2^{i}},\frac{n}{2^{N}}\right)-\frac{f\left(\frac{2j-2}{2^{i}},\frac{n}{2^{N}}\right)+f\left(\frac{2j}{2^{i}},\frac{n}{2^{N}}\right)}{2}.$$

In (27), the hat functions *y_j,i_* (*x*_1_) are exactly the same as those for the 1D case, generated by the same universal deep network as shown in Fig. 3 if *N* = 3. In the 1D case, the weights *a*_j,i_ are scalars, directly evaluated from the target function *f* (*x*). The formula for the weights (24) still applies in the 2D case. The only difference is that the second variable *x*_2_ needs to be fixed to a value *x*_2_ = *n*/2*^N^* as shown in (28). The final expression for the 2D piecewise approximation $\hat{f}(x_{1},x_{2})$ is achieved by linearly interpolating the functions $\hat{f}\left(x,n∕2^{N}\right)$ calculated in (27) as

(29) $$\hat{f}\;(x_{1},x_{2})=\sum_{n=1}^{2^{N}-1}\hat{f}\left(x_{1},\frac{n}{2^{N}}\right)y_{n,N}\;(x_{2}).$$

Eq. (29) is almost the same as Eq. (1) and can be implemented by 1 layer of the network.

### E. EXTENSION TO A d-D DEEP NETWORK WITH N HIDDEN LAYERS

If the target function *f* (*x*_1_, *x*_2_, …, *x_d_*) is *d*-dimensional (*d*-D), its deep network approximation $\hat{f}(x_{1},x_{2},\ldots ,x_{d})$ can be obtained like the 2D case presented in Part D.

Let *f* (*x*_1_, *x*_2_,…, *x_d_*) be sampled uniformly at the knots (*m*_1_ /2*^N^* , *m*_2_/2*^N^* ,…, *m_d_*/2*^N^*) with *m_k_* = 0, 1, 2, …, 2*^N^* and *k* = 1, 2, …, *d*.

We apply the universal deep network approximation to the variable *x*_1_. Eq. (27) becomes

(30) $$\hat{f}\left(x_{1},\frac{m_{2}}{2^{N}},\ldots ,\frac{m_{d}}{2^{N}}\right)\;\;=\left[f\left(1,\frac{m_{2}}{2^{N}},\ldots ,\frac{m_{d}}{2^{N}}\right)-f\left(0,\frac{m_{2}}{2^{N}},\ldots ,\frac{m_{d}}{2^{N}}\right)\right]x_{1}\;\;+f\left(0,\frac{m_{2}}{2^{N}},\ldots ,\frac{m_{d}}{2^{N}}\right)\;\;+\sum_{i=1}^{N}\sum_{j=1}^{2^{i-1}}a_{j,i}(\frac{m_{2}}{2^{N}},\ldots ,\frac{m_{d}}{2^{N}})y_{j,i}\;(x_{1})$$

where

(31) $$a_{j,i}\left(\frac{m_{2}}{2^{N}},\ldots ,\frac{m_{d}}{2^{N}}\right)\;\;=f\left(\frac{2j-1}{2^{i}},\frac{m_{2}}{2^{N}},\ldots ,\frac{m_{d}}{2^{N}}\right)\;\;-\frac{f\left(\frac{2j-2}{2^{i}},\frac{m_{2}}{2^{N}},\ldots ,\frac{m_{d}}{2^{N}}\right)+f\left(\frac{2j}{2^{i}},\frac{m_{2}}{2^{N}},\ldots ,\frac{m_{d}}{2^{N}}\right)}{2}.$$

The final expression for the *d*-D piecewise approximation $\hat{f}(x_{1},x_{2},\ldots ,x_{d})$ is achieved by linearly interpolating the functions $\hat{f}\left(x_{1},m_{2}∕2^{N},\ldots ,m_{d}∕2^{N}\right)$ calculated in (29) as

(32) $$\hat{f}\;(x_{1},x_{2},\ldots ,x_{d})\;\;=\sum_{m_{d}=1}^{2^{N}-1}\cdots \sum_{m_{2}=1}^{2^{N}-1}\hat{f}\left(x_{1},\frac{m_{2}}{2^{N}},\ldots ,\frac{m_{d}}{2^{N}}\right)\;\;\times y_{m_{2},N}\;(x_{2})\ldots y_{m_{d},N}\;(x_{d}).$$

There are *d* — 1 layers of summations in (32). Therefore, (32) can be implemented by *d* — 1 layers of the interpolation network.

## III. RESULTS

In this section, we present 2 numerical examples to use the proposed deep network architecture to approximate the target functions.

**FIGURE 10.**
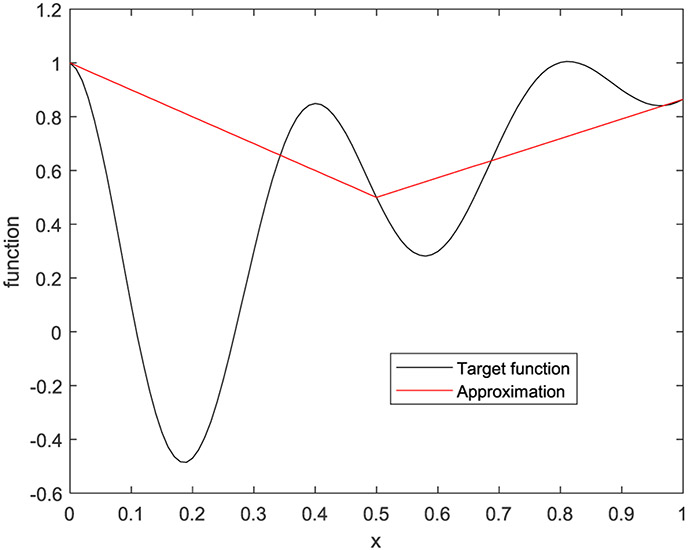
The target function is in black, and the piecewise approximation is in red using 1 hidden layer.

**FIGURE 11.**
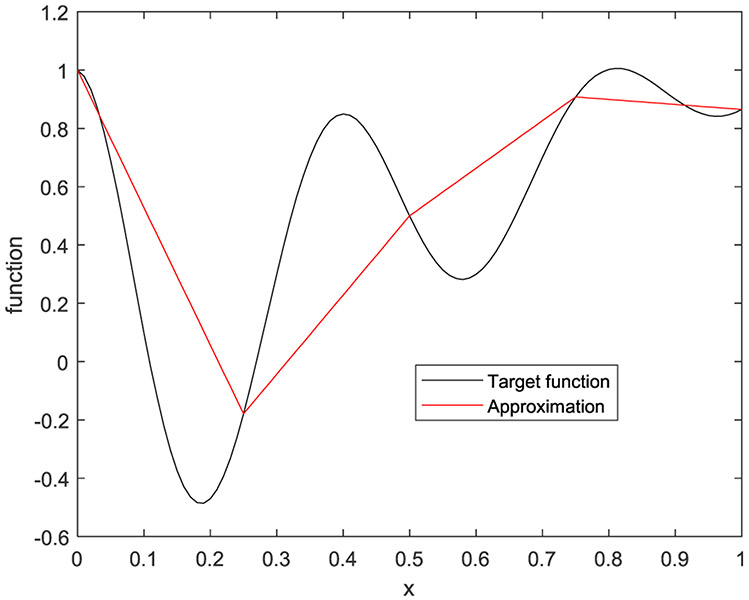
The target function is in black, and the piecewise approximation is in red using 2 hidden layers.

**FIGURE 12.**
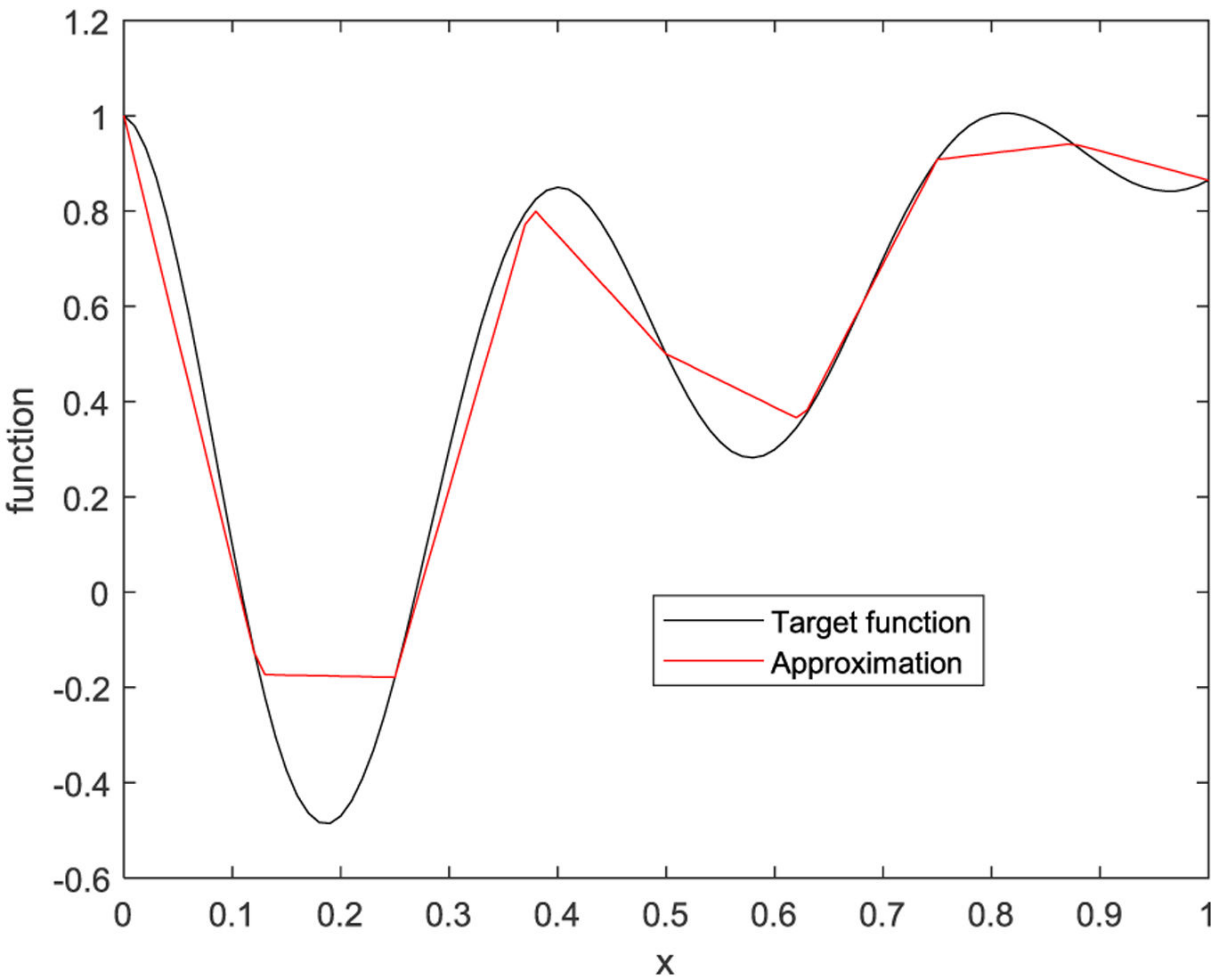
The target function is in black, and the piecewise approximation is in red using 3 hidden layers.

**FIGURE 13.**
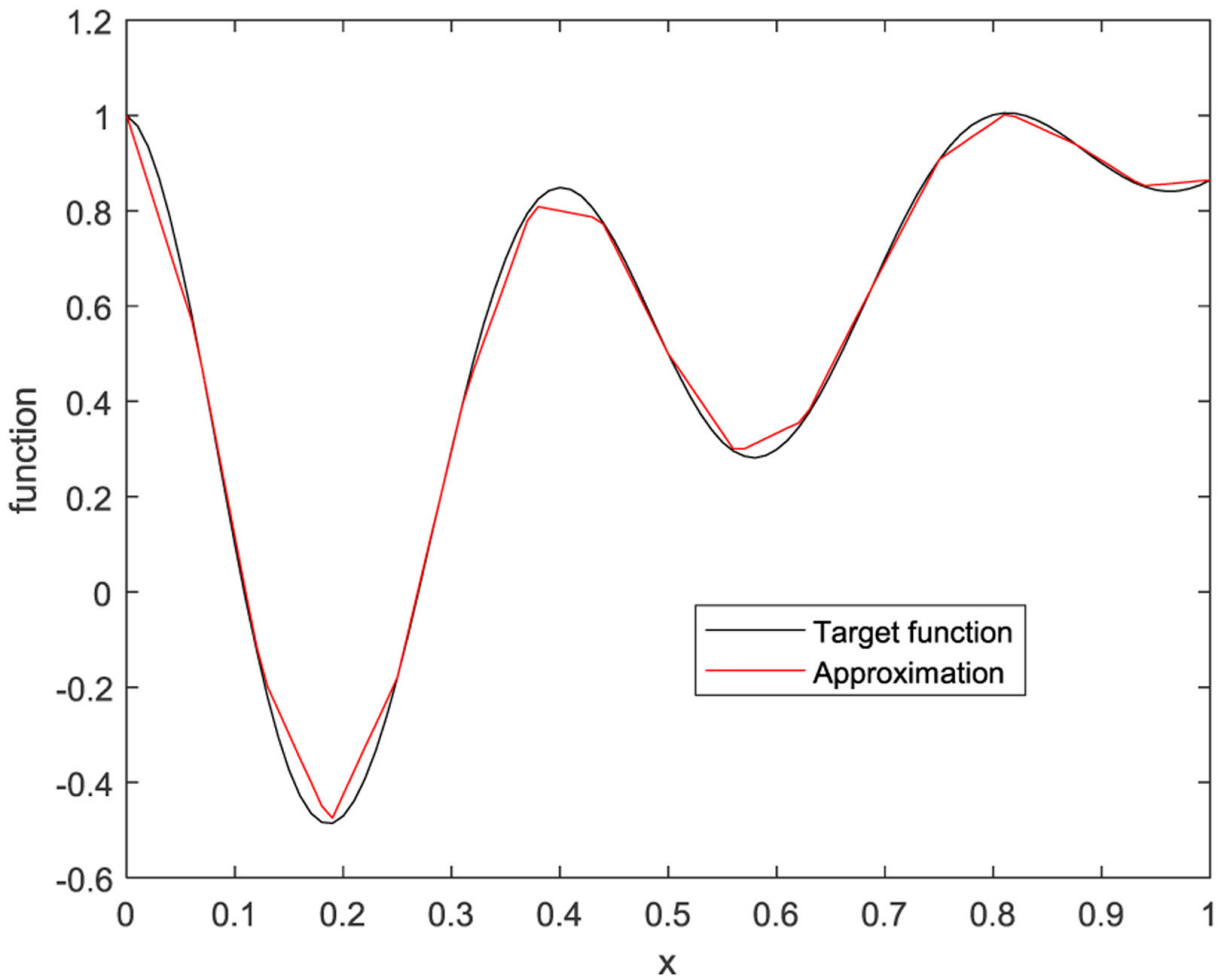
The target function is in black, and the piecewise approximation is in red using 4 hidden layers.

**FIGURE 14.**
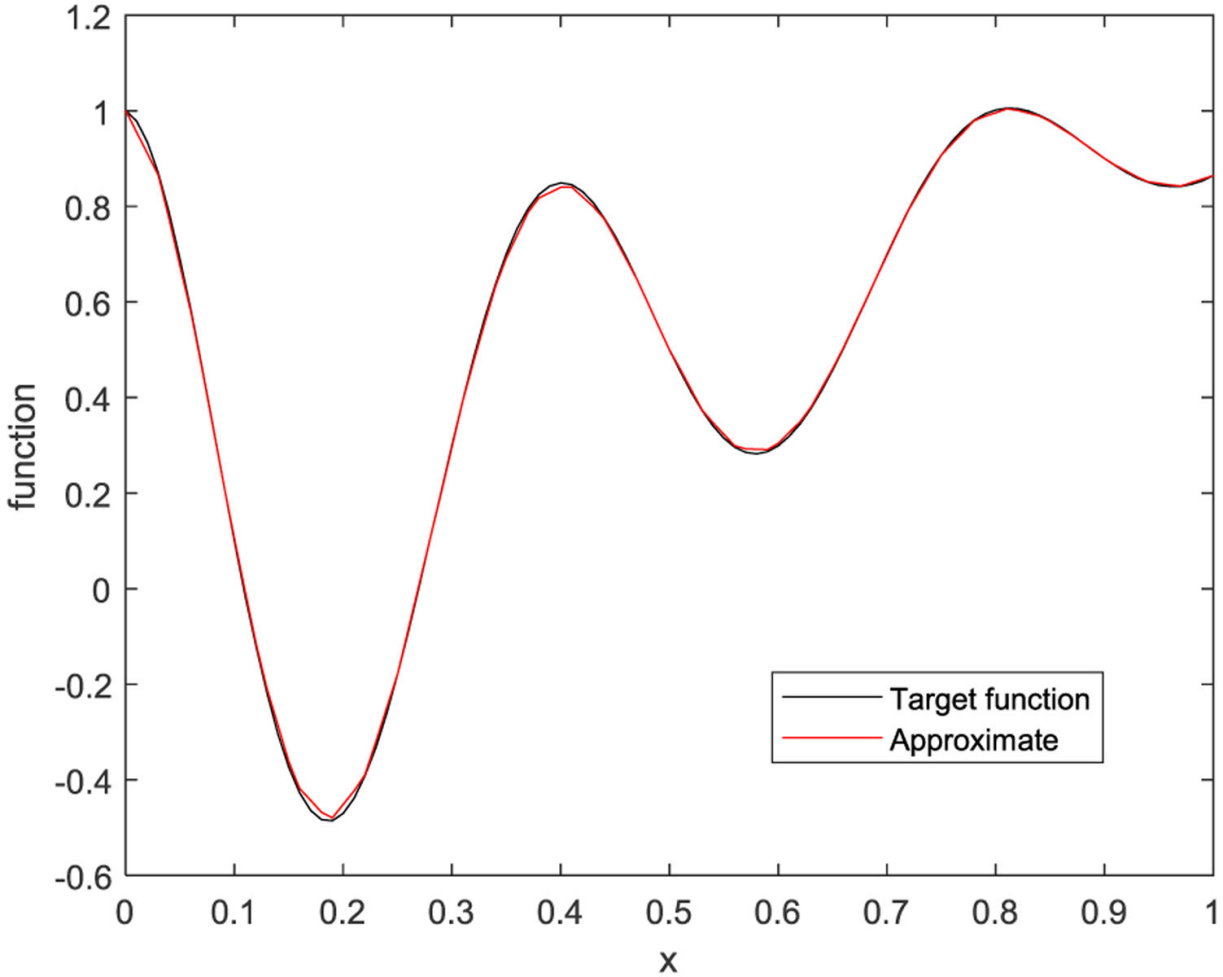
The target function is in black, and the piecewise approximation is in red using 5 hidden layers.

In the first example, the target function is a 1D function defined by

(33) $$f\;(x)=e^{-2x}cos\;(5\pi x)+x$$

on [0, 1]. Five cases are presented with the number of hidden layers being 1, 2, 3, 4, and 5, respectively. The corresponding results are shown in Figs. 10 to 14, respectively.

**FIGURE 15.**
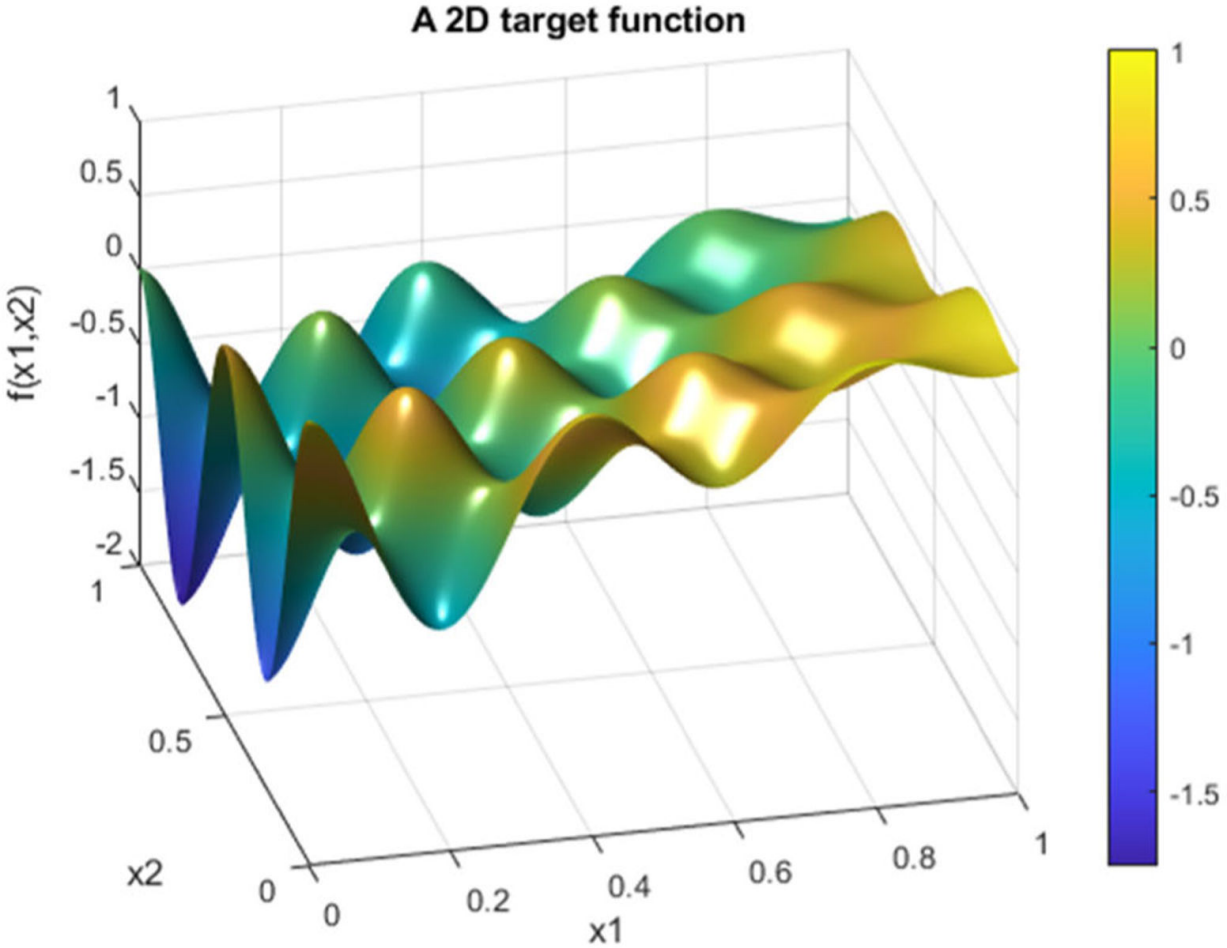
A mesh surface display of the 2D target function defined in (34).

**FIGURE 16.**
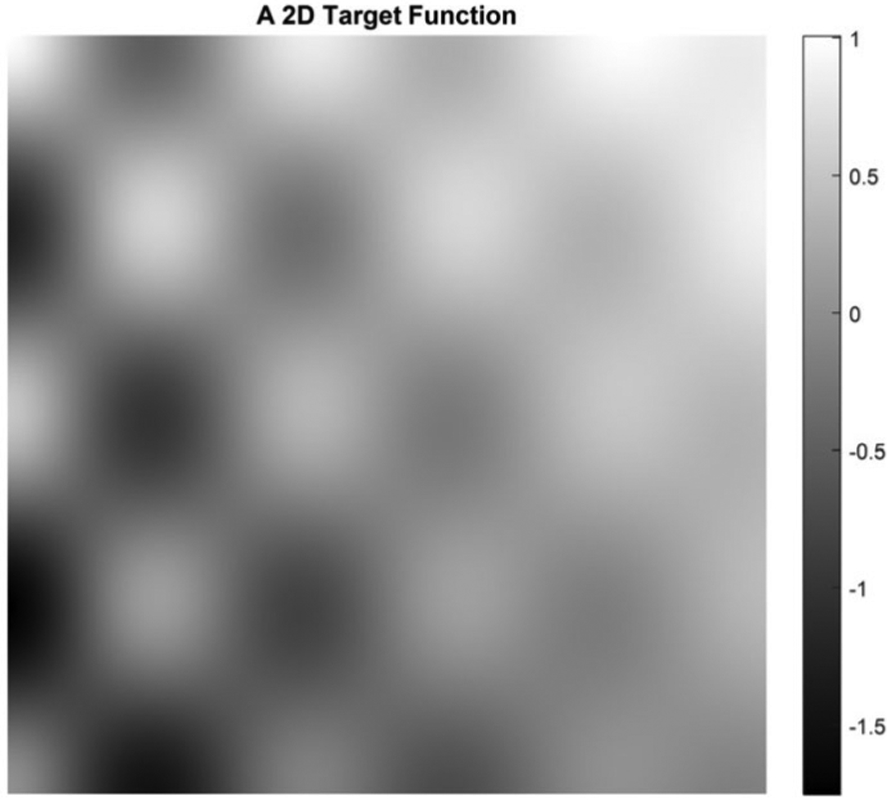
A gray scale display of the 2D target function defined in (34).

In the second example, the target function is a 2D function defined by

(34) $$f\;(x_{1},x_{2})=e^{-2x_{1}}cos\;(5\pi x_{1})\;cos\;(4\pi x_{2})+x_{1}-x_{2}$$

on [0, 1] × [0, 1]. Five cases are presented with the number of hidden layers in the universal deep network associated with the variable *x*_1_ being 1, 2, 3, 4, and 5, respectively. The function defined in (34) is displayed as the mesh surface in Fig. 15. An alternative way to display a 2D function is using the gray scale. A gray scale display of the 2D target function (34) is given in Fig. 16.

**FIGURE 17.**
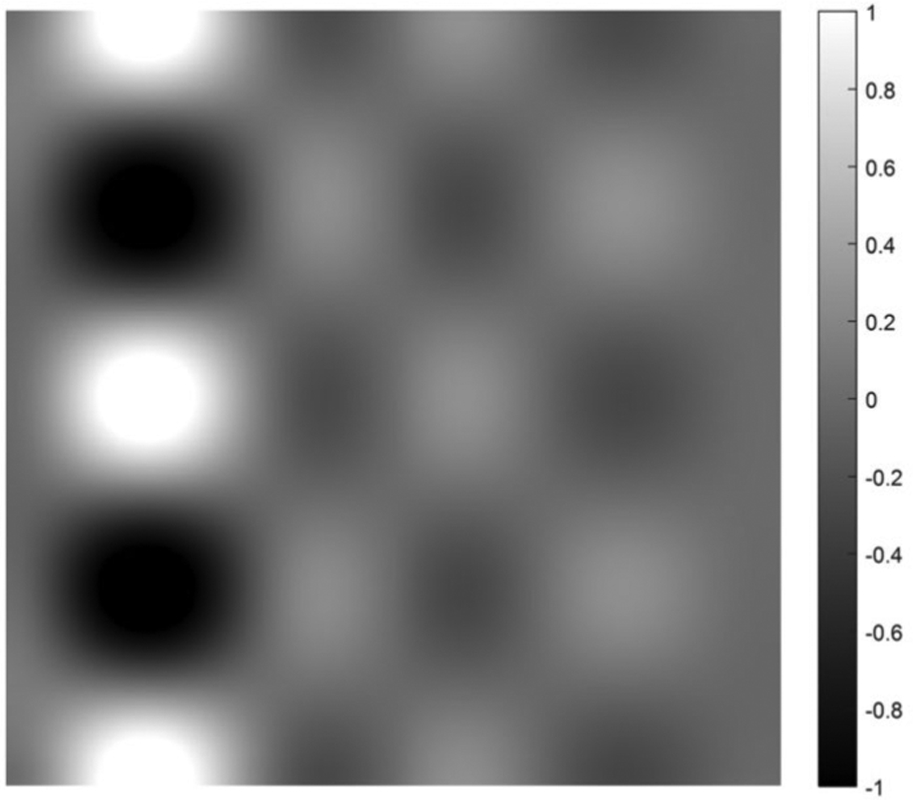
A gray scale display of the approximation error between the approximation using 1 hidden layer and the 2D target function.

**FIGURE 18.**
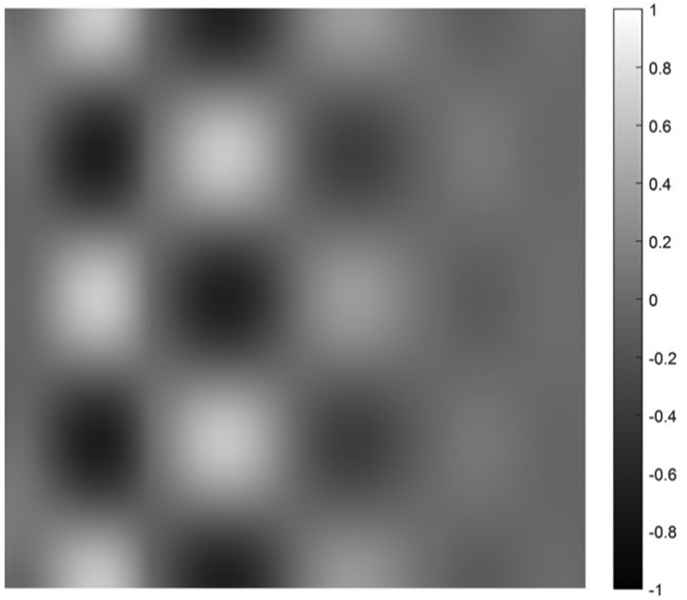
A gray scale display of the approximation error between the approximation using 2 hidden layers and the 2D target function.

**FIGURE 19.**
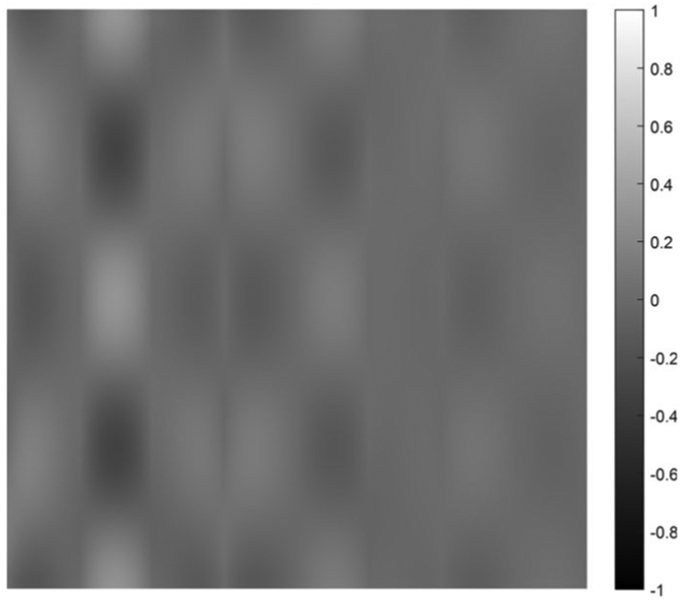
A gray scale display of the approximation error between the approximation using 3 hidden layers and the 2D target function.

**FIGURE 20.**
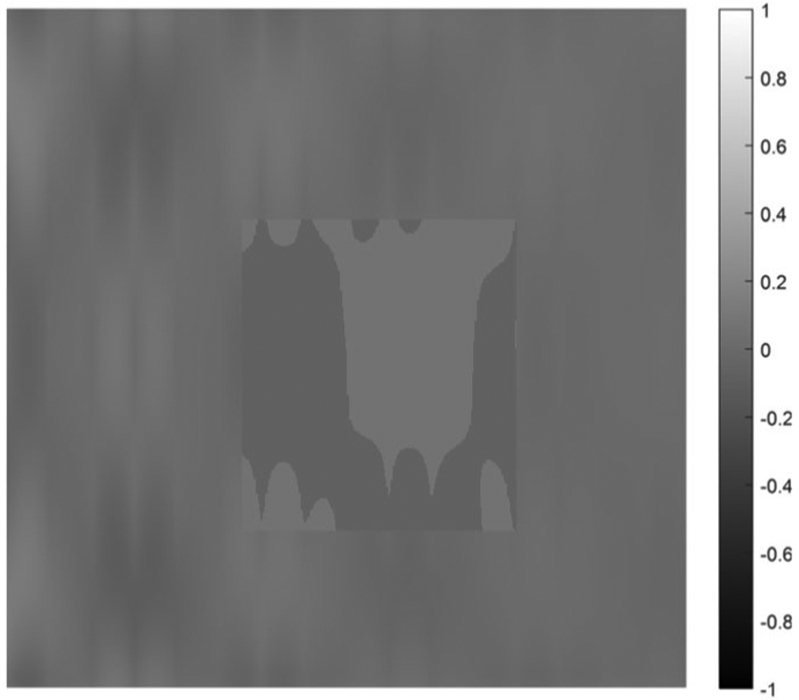
A gray scale display of the approximation error between the approximation using 4 hidden layers and the 2D target function.

**FIGURE 21.**
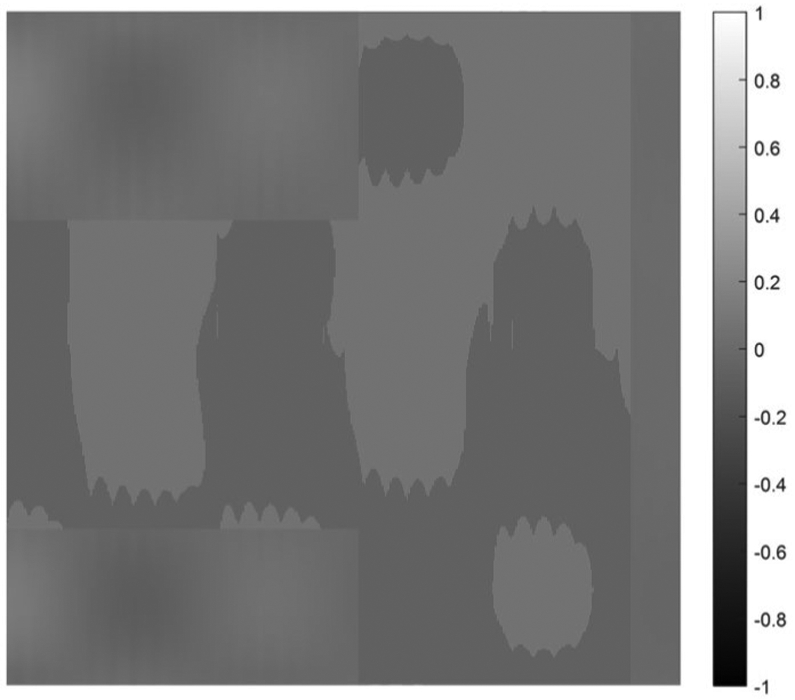
A gray scale display of the approximation error between the approximation using 5 hidden layers and the 2D target function.

Since the mesh surface plots are difficult to see small differences, the gray scale images of the results of the approximation errors $\hat{f}\;(x_{1},x_{2})-f(x_{1},x_{2})$ are shown in Figs. 17 to 21, respectively. For a good approximation, the values in the error image should be very close to zero.

The purpose of these two examples is to verify the feasibility of the proposed universal deep network architecture (see Fig. 3) and its associated formula (24) for the weights. The depth *N* of the universal deep network is determined by the target function *f* and the pre specified accuracy. We can start with a small *N*. If the approximation error is too large, we then increase *N* until the approximation errors are in the satisfactory range. When the depth *N* is increase by 1, the number of function sampling knots is doubled.

## IV. DISCUSSION

In general machine learning, when we have the input and output data pairs, a training algorithm is applied for mapping the relationship between the input and output, to obtain the weights in the neural network. If we know the analytical formula of the training function, we do not need to use a training algorithm to obtain the weights.

We now discuss some strengths and weaknesses of our work in this paper. One strength is that for any continuous target function, we can construct a deep network to approximate the target function. Another strength is that the proposed deep network has a universal architecture that is independent from the target function. The third strength is that the weights of this universal deep network can be calculated by an explicit formula. Therefore, no training is required. To extend the proposed universal deep network from 1D function approximation (associated with the variable *x*_1_) to *d*-D function approximation, one only needs to add one post interpolation layer for each additional variable (such as *x*_2_).

The weaknesses of the proposed work are as follows. The main motivation for using a deep network instead of a shallow network is the hope that the deep network is able to reach the same approximation accuracy with fewer weights. Unfortunately, the proposed deep network has the same approximation accuracy using the same number of weights as the shallow network. It is unknown to us whether it is possible to develop a more efficient universal deep network for the same approximation accuracy.

It can be readily verified that the deep network approximation (25) is the same as the shallow network approximation (3) if they use the same number of knots. In fact, these two approximations have the same values at the sampling knots as the target function and they take the linear interpolated values between the knots.

Our proposed deep network architecture is different from the standard perceptron architecture, which is essentially the composite affine transform with activation functions. It is unclear to us that there is an explicit formula for the weights in the standard deep perceptron network.

## V. CONCLUSION

We have developed a deep network architecture, which produces left-half and right-half hat functions with different widths and different locations. The sum of a pair of the left-half and right-half hat functions results in a full hat function. At each hidden layer, the hat functions partition the interval [0, 1]. From one layer to the next layer, the number of hat functions doubles, and the width of hat functions halves. The architecture follows the Gray code order.

If the number of the hidden layers is *N*, the weights are calculated from the values of the target function at the uniform knots on [0, 1]: *j*/2*^N^* for *j* = 0, 1, 2, 3, …, 2*^N^*. The general formula for the weights is given by (24). The final expression of the approximation function is given by (25). This 1D universal deep network architecture can be readily extended to *d*-D functions, by adding a post linear interpolation layer for each additional variable.

## Figures and Tables

**TABLE 1.** Gray codes and corresponding subscripts of function *g*.

1-bit Gray code; subscript of *g* for the 1st hidden layer	2-bit Gray code; subscript of *g* for the 2nd hidden layer	3-bit Gray code; subscript of *g* for the 3rd hidden layer
0; g_0_(*x*)	00; g_0_(g_0_(*x*))	000; g_0_(g_0_(g_0_(*x*)))
1; g_1_(*x*)	01; g_1_(g_0_(*x*)	001; g_1_(g_0_(g_0_(*x*)))
	11; g_1_(g_1_(*x*))	011; g_1_(g_1_(g_0_(*x*)))
	10; g_0_(g_1_(*x*))	010; g_0_(g_1_(g_0_(*x*)))
		110; g_0_(g_1_(g_1_(*x*)))
		111; g_1_(g_1_(g_1_(*x*)))
		101; g_1_(g_0_(g_1_(*x*)))
		100; g_0_(g_0_(g_1_(*x*)))

## References

[1] Tappert CC, “Who is the father of deep learning?” in Proc. Int. Conf. Comput. Sci. Comput. Intell. (CSCI), 12. 2019, pp. 343–348, doi: 10.1109/CSCI49370.2019.00067.

[2] Ivakhnenko AG and Lapa VG, Cybernetics and Forecasting Techniques. New York, NY, USA: American Elsevier Publishing Co., 1967.

[3] Rumelhart DE, Hinton GE, and Williams RJ, “Learning representations by back-propagating errors,” Nature, vol. 323, pp. 533–536, 10. 1986, doi: 10.1038/323533a0.

[4] LeCun Y, Boser B, Denker JS, Henderson D, Howard RE, Hubbard W, and Jackel LD, “Backpropagation applied to handwritten zip code recognition,” Neural Comput., vol. 1, no. 4, pp. 541–551, 1989, doi: 10.1162/neco.1989.1.4.541.

[5] Hinton G, “Deep belief networks,” Scholarpedia, vol. 4, no. 5, p. 5947, 2009, doi: 10.4249/scholarpedia.5947.

[6] Lin Y, Jiang Z, Gu J, Li W, Dhar S, Ren H, Khailany B, and Pan DZ, “DREAMPlace: Deep learning toolkit-enabled GPU acceleration for modern VLSI placement,” IEEE Trans. Comput.-Aided Design Integr. Circuits Syst, vol. 40, no. 4, pp. 748–761, 4. 2021, doi: 10.1109/TCAD.2020.3003843.

[7] L’Heureux A, Grolinger K, Elyamany HF, and Capretz MAM, “Machine learning with big data: Challenges and approaches,” IEEE Access, vol. 5, pp. 7776–7797, 2017, doi: 10.1109/ACCESS.2017.2696365.

[8] Hornik K, Stinchcombe M, and White H, “Multilayer feedforward networks are universal approximators,” Neural Netw., vol. 2, no. 5, pp. 359–366, 1989. [Online]. Available: http://cognitivemedium.com/magic_paper/assets/Hornik.pdf

[9] Sci JH, “Relu deep neural networks and linear finite elements,” J. Comput. Math, vol. 38, no. 3, pp. 502–527, 6. 2020, doi: 10.4208/jcm.1901-m2018-0160.

[10] Yarotsky D, “Error bounds for approximations with deep ReLU networks,” Neural Netw., vol. 94, pp. 103–114, 10. 2017, doi: 10.1016/j.neunet.2017.07.002. 28756334

[11] Anthony M and Bartlett PL, Neural Network Learning: Theoretical Foundations. Cambridge, U.K.: Cambridge Univ. Press, 2009.

[12] Linz P and Wang R, Exploring Numerical Methods: An Introduction to Scientific Computing Using MATLAB. Sudbury, MA, USA: Jones Bartlett Publishers, 2003.

[13] Lucal HM, “Arithmetic operations for digital computers using a modified reflected binary code,” IRE Trans. Electron. Comput, vol. EC-8, no. 4, pp. 449–458, 12. 1959, doi: 10.1109/TEC.1959.5222057.

[14] He N, Shi D, and Chen T, “Self-triggered model predictive control for networked control systems based on first-order hold,” Int. J. Robust Nonlinear Control, vol. 28, no. 4, pp. 1303–1318, 3. 2018, doi: 10.1002/rnc.3953.

